# Interpreting Acupuncture‐Specific Neuroplasticity in Poststroke Motor Recovery: Statistical and Methodological Considerations

**DOI:** 10.1002/cns.71265

**Published:** 2026-11-11

**Authors:** Han Xue, Jun Hu

**Affiliations:** ^1^ School of Rehabilitation Science Shanghai University of Traditional Chinese Medicine Shanghai China; ^2^ Shanghai Second Rehabilitation Hospital Shanghai China

Dear Editor,

1

We read with great interest the recent randomized multimodal MRI study by Yu et al. [1], which integrated dynamic functional network analysis and voxel‐based morphometry to investigate neuroplastic changes associated with acupuncture during poststroke motor recovery. The study provides valuable longitudinal neuroimaging observations. However, we believe that the interpretation of these findings as evidence of acupuncture‐specific neuroplasticity requires further statistical clarification.

The principal concern is that a significant pre–post change in the true‐acupoint (TA) group, together with a non‐significant change in the sham‐acupoint (SA) group, does not by itself establish a significant difference in treatment effects between groups [2, 3]. In a randomized two‐group pre–post design, treatment specificity requires direct comparison of longitudinal change between groups (ΔTA − ΔSA), for example through an appropriately specified group × time interaction. Notably, the authors explicitly acknowledge the absence of a statistically significant group × time interaction, whereas the Methods primarily describe within‐group paired analyses [1]. This creates an important interpretive inconsistency: despite the absence of a statistically significant group × time interaction supporting differential longitudinal effects between groups, the article repeatedly characterizes TA as producing “superior,” motor recovery and “specific,” neuroplastic changes, and further states that the neural reorganization observed only in the TA group confirms effects beyond non‐specific or placebo responses. A non‐significant interaction does not establish equivalence between TA and SA; however, it also does not provide confirmatory evidence that TA produced a greater treatment‐related change. Accordingly, these findings are more appropriately interpreted as TA‐associated longitudinal changes unless direct between‐group comparisons support treatment specificity.

These considerations directly affect both the clinical and neuroimaging interpretations. Clinically, the authors report significant improvements in FMA and NIHSS scores in both groups, whereas Brunnstrom scores improved significantly only in the TA group, and subsequently describe motor recovery as superior with TA [1]. However, significance in one group and non‐significance in another do not demonstrate a between‐group difference in treatment response. The same issue applies to the neuroimaging findings. TA was associated with reduced DMN disjointedness (*p*_FDR_ = 0.041) and a trend toward reduced flexibility (*p*_FDR_ = 0.094), whereas no corresponding significant changes were detected within the SA group. Similarly, significant GMV increases were identified only within the TA group [1]. These observations support TA‐associated longitudinal changes, but without direct TA‐versus‐SA comparisons of change, they do not establish that the functional or structural alterations are specific to true‐acupoint acupuncture. This distinction is particularly relevant given the unequal final imaging sample sizes (TA, *n* = 30; SA, *n* = 16), which may confer different statistical power for detecting within‐group effects.

Several numerical inconsistencies in the reported clinical data also warrant clarification. The baseline FMA‐Lower score for the TA group is reported as 26.60 ± 7.92 in Table 1 of Yu et al. [1] but as 27.60 ± 7.92 in their Table 2. More notably, the post‐treatment total FMA score in the SA group is listed as 59.40 ± 11.54, despite a baseline value of 68.25 ± 32.46 and the accompanying text describing significant improvement in FMA (*p* = 0.015). The value 59.40 ± 11.54 is also identical to the mean age reported for the TA group in Table 1 of Yu et al. [1], raising the possibility of a transcription or typesetting error. Because these clinical outcomes contribute to the interpretation of motor recovery and its associations with neuroimaging changes, clarification of the underlying values would help ensure accurate interpretation.

**TABLE 1:** Demographics, clinical variables of study participants.

Demographics	TA group (*n* = 30)	SA group (*n* = 16)	*p*	*t*
Age (SD)	59.40 ± 11.54	61.13 ± 7.78	0.602	−0.525
Men/women	21/9	13/3	0.635	—
Course of disease (SD)	15.30 ± 11.78	15.56 ± 12.84	0.334	−0.336
Dominant hand (right)	30	16	—	—
FMA (SD)	67.10 ± 26.59	68.25 ± 32.46	0.900	−0.126
FMA‐Lower (SD)	26.60 ± 7.92	26.88 ± 7.94	0.913	−0.110
FMA‐Upper (SD)	40.47 ± 23.73	41.44 ± 25.23	0.900	−0.126
NIHSS(SD)	3.43 ± 2.55	4.50 ± 3.45	0.253	−1.158
Brunnstrom‐Upper (SD)	4.13 ± 2.05	4.13 ± 2.05	1.000	0.000
Brunnstrom‐Lower (SD)	4.57 ± 1.36	4.56 ± 1.62	0.993	0.009

Abbreviations: FMA, Fugl‐Meyer Assessment; NIHSS, National Institutes of Health Stroke Scale; SA, sham‐acupoint; SD, standard deviation; TA, true‐acupoint.

**TABLE 2:** Treatment outcomes of participants.

Clinical scales (SD)	TA group (*n* = 30)				SA group (*n* = 16)			
	Before	After	*p*	*t*	Before	After	*p*	*t*
FMA	67.10 ± 26.59	76.63 ± 24.41	< 0.001	−6.230	68.25 ± 32.46	59.40 ± 11.54	0.015	−2.760
FMA‐Lower	27.60 ± 7.92	30.17 ± 5.23	< 0.001	−3.864	26.88 ± 7.94	28.88 ± 6.34	0.012	−2.877
FMA‐Upper	40.47 ± 23.73	46.73 ± 21.09	< 0.001	−4.716	41.44 ± 25.23	46.38 ± 22.65	0.035	−2.318
NIHSS	3.43 ± 2.58	2.30 ± 2.42	< 0.001	4.852	4.50 ± 3.45	2.63 ± 2.74	< 0.001	4.496
Brunnstrom‐Upper	4.13 ± 2.05	4.80 ± 1.66	< 0.001	−3.959	4.13 ± 2.00	4.98 ± 1.83	0.333	−1.000
Brunnstrom‐Lower	4.57 ± 1.36	5.30 ± 1.04	< 0.001	−3.717	4.56 ± 1.62	4.88 ± 1.49	0.237	−1.232

*Note:* Bold values in Table 2 indicate statistical significance (*p* < 0.05).

Abbreviations: FMA, Fugl‐Meyer Assessment; NIHSS, National Institutes of Health Stroke Scale; SA, sham‐acupoint; SD, standard deviation; TA, true‐acupoint.

The description of the acupuncture intervention also appears to contain inconsistencies that merit clarification. In section 2.5 of the original article [1], the TA protocol lists Quchi (LI11), Neiguan (SJ5), Hegu (SJ5), Zusanli (ST36), Sanyinjiao (SP6), and Yanglingquan (GB34), whereas figure 1 of Yu et al. [1] lists LI11, SJ5, LI4, ST36, SP6, and LR3. Figure 2 of the original article [1], in turn, depicts LI11, SJ5, LI4, ST36, SP6, and GB34. These discrepancies make it unclear which acupoints were actually administered. Because the study specifically seeks to distinguish true‐acupoint from sham‐acupoint effects, an unambiguous description of the intervention is essential for reproducibility and for interpreting claims of treatment specificity.

We therefore encourage the authors to clarify which outcomes were included in the reported group × time analysis and to provide the corresponding effect estimates, 95% confidence intervals, and *p* values. For neuroimaging outcomes not assessed using a direct interaction analysis, TA–SA contrasts of longitudinal change, with appropriate correction for multiple comparisons, would help determine whether the observed alterations in DMN dynamics and GMV differ between interventions. Clarification of the reported clinical values and the exact acupoints administered would also improve the transparency and reproducibility of the study. Such clarification would preserve the value of the reported multimodal neuroimaging findings while more clearly distinguishing TA‐associated longitudinal changes from statistically supported treatment‐specific differences relative to sham acupuncture.

## Conflicts of Interest

The authors declare no conflicts of interest.

## Data Availability

Data sharing is not applicable to this article as no datasets were generated or analyzed during the current study.
